# Dasatinib and Doxorubicin Treatment of Sarcoma Initiating Cells: A Possible New Treatment Strategy

**DOI:** 10.1155/2016/9601493

**Published:** 2015-12-15

**Authors:** Ninna Aggerholm-Pedersen, Christina Demuth, Akmal Safwat, Peter Meldgaard, Moustapha Kassem, Boe Sandahl Sorensen

**Affiliations:** ^1^Department of Oncology, Aarhus University Hospital, 8000 Aarhus C, Denmark; ^2^Department of Clinical Biochemistry, Aarhus University Hospital, 8000 Aarhus C, Denmark; ^3^Laboratory for Molecular Endocrinology (KMEB), Department of Endocrinology, Odense University Hospital, 5000 Odense, Denmark; ^4^Danish Stem Cell Center (DanStem), Panum Institute, University of Copenhagen, 2200 Copenhagen, Denmark

## Abstract

*Background*. One of the major challenges affecting sarcoma treatment outcome, particularly that of metastatic disease, is resistance to chemotherapy. Cancer-initiating cells are considered a major contributor to this resistance.* Methods*. An immortalised nontransformed human stromal (mesenchymal) stem cell line hMSC-TERT4 and a transformed cell line hMSC-TERT20-CE8, known to form sarcoma-like tumours when implanted in immune-deficient mice, were used as models. Receptor tyrosine kinase (RTK) activation was analysed by RTK arrays and cellular viability after tyrosine kinases inhibitor (TKI) treatment with or without doxorubicin was assessed by MTS assay.* Results*. Initial results showed that the hMSC-TERT4 was more doxorubicin-sensitive while hMSC-TERT20-CE8 was less doxorubicin-sensitive evidenced by monitoring cell viability in the presence of doxorubicin at different doses. The epidermal growth factor receptor (EGFR) was activated in both cell lines. However hMSC-TERT20-CE8 exhibited significantly higher expression of the EGFR ligands. EGFR inhibitors such as erlotinib and afatinib alone or in combination with doxorubicin failed to further decrease cell viability of hMSC-TERT20-CE8. However, inhibition with the TKI dasatinib in combination with doxorubicin decreased cell viability of the hMSC-TERT20-CE8 cell line.* Conclusion*. Our results demonstrate that dasatinib, but not EGFR-directed treatment, can decrease cell viability of stromal cancer stem cells less sensitive to doxorubicin.

## 1. Background

Sarcomas are suggested to develop from stromal (also known as mesenchymal) stem cells with acquired genetic mutations leading to cellular transformation [1, 2]. It is generally believed that the poor clinical outcome is directly related to chemotherapy-resistant cancer-initiating stem cells. The standard first line treatment of patients with metastatic soft tissue sarcoma (STS) is doxorubicin. However, most metastatic sarcomas have intrinsic resistance or will acquire resistance to doxorubicin. Unfortunately, no significant changes have occurred during the past 20 years regarding treatment options available for patients with metastatic STS [3] and the median overall survival for these patients continues to be around 2 years [4].

Activation of critical survival pathways in stromal cancer-initiating cells through receptor tyrosine kinases (RTKs) signaling contributes to chemotherapy-resistance in sarcomas as RTKs signaling is important for maintenance of cell survival [5].

Combining chemotherapy with RTK-targeted treatment presents an advantage for targeting cellular resistance against doxorubicin. This approach has been successful in overcoming tumour cell resistance to doxorubicin in small cell sarcoma cell lines [6]. In spite of this initial success, it is still unclear which RTKs to target and if a combined treatment modality is effective. Few models are available for studying the biology of sarcoma cancer-initiating cells in vitro. We have developed an in vitro model for sarcoma based on telomerised human stromal cells [7]. In long term cultures, these cells acquired a transformed phenotype [2, 8]. Histological analysis of the tumour formed in immune deficient mice revealed a sarcoma phenotype [8, 9]. Employing this cell model, our aim was to identify RTKs activated in stromal cancer stem cells and investigate if chemoresistance can be overcome by a simultaneous targeting of relevant RTKs.

## 2. Materials and Methods

### 2.1. Tumour Model and Cell Culture

We used a cell model for sarcoma stem cell that has been established from a human bone marrow stromal (mesenchymal) stem cell (hMSC). The parental hMSC was immortalized by retroviral transduction of human telomerase reverse transcriptase (hTERT) gene and named hMSC-TERT [7, 10]. The parental nontumorigenic hMSC-TERT4 has the ability of extensive proliferation in addition to its capacity for multilineage differentiation but it does not have the ability to form tumours [8]. The hMSC-TERT cells spontaneously acquired the transformed phenotype during long term culture in vitro as evidenced by the ability to form tumours in nude mice [2]. A clonal population derived from the parental hMSC-TERT designated hMSC-TERT20-CE8 was chosen [8]. The derivation and characterization of nontransformed cell line hMSC-TERT and the clone hMSC-TERT20-CE8 have been described previously. These cells have the potential to differentiate to specific lineages of mesenchymal tissue under well-defined culturing conditions [2, 8]. The transformed cell lines form sarcoma-like tumours when implanted in immune deficient mice in vivo [2, 9]. Both cell lines were passaged in minimal essential media (MEM) (Gibco, Life Technologies) supplemented with 1% L-glutamine (Gibco, Life Technologies), 10% fetal bovine serum (Gibco, Life Technologies), and 1% Penicillin-Streptomycin (Gibco, Life Technologies). Cells were incubated in 5% CO_2_ humidified atmosphere at 37°C.

### 2.2. Cell Treatments and Viability Assay

To assess cell viability, we used CellTiter 96 Aqueous Nonradioactive Cell Proliferation Assay (MTS) (Promega) following manufacturer's instructions. Erlotinib, afatinib, and dasatinib were purchased as 10 mM stocks dissolved in DMSO (Selleckchem). Doxorubicin (Accord) 2 mg/mL was diluted in 10 mL NaCl 9 mg/mL.

Adherent cells were detached using Trypsin-EDTA (0.25%, Gibco, Life Technologies) for 2 min. After detachment, 10 mL MEM media were added and cells were centrifuged at 1800 RPM for 3 min. Cells were resuspended in MEM media and plated in 96-well plates at a concentration of 5000 cells/well. After 24 hours of incubation, the cells were treated with different concentrations of TKIs (0.01–5 *μ*M), doxorubicin (5–100 nM), or DMSO alone for 72 h. After the treatment for the indicated time, MTS was added to each well and incubated at 37°C for 1 to 4 h. The MTS containing medium was transferred to a new 96-well plate and the absorbance at 492 nm was measured (Background 690 nm). MTS control MEM media without cells were added to each plate. Results were expressed as fold changes compared to the untreated control.

### 2.3. Receptor Tyrosine Kinase (RTK) Activation

Cellular activation of forty-nine different human RTKs was assessed using the Human Phospho-Receptor Tyrosine Kinase Array Kit (R&D Systems) following manufacturer's instructions.

### 2.4. RNA Extraction and qPCR

The cells were harvested using a cell scraper, centrifuged at 2000 rpm for 5 min, and resuspended in Buffer RLT (Qiagen). RNA was extracted using the RNeasy Mini Kit on the QIAcube platform (Qiagen). RNA was eluted in RNase free water for further analysis. The amount of purified RNA was quantified by the Nanodrop 2000c (Thermo Scientific).

Complementary DNA (cDNA) was synthesized from 100 ng total RNA using 50 U MuLV Reverse Transcriptase (Applied Biosystems), 1X PCR buffer (Applied Biosystems), 6.3 mM MgCl_2_ (Applied Biosystems), 1 mM of each of dATP, dTTP, dGTP, and dCTP (VWR), 2.5 *μ*M Oligod (T_16_) Primer (DNA Technology), and 20 U RNase inhibitor (Applied Biosystems). The final reaction was diluted to a total volume of 20 *μ*L.

Quantitative PCR (qPCR) was performed in a 10 *μ*L reaction volume containing 5 *μ*L LightCycler 480 SYBR Green I Master (Roche Diagnostics, Mannheim), 3 *μ*L RNase free H_2_O, 0.5 *μ*L forward primer (5 pm/*μ*L), 0.5 *μ*L reverse primer (5 pm/*μ*L), and 1 *μ*L cDNA. The PCR reaction was as follows: 95°C for 10 min, 50 cycles of 95°C for 10 sec, specific annealing temperature for 20 sec, and 72°C for 5 sec. Melting curves were produced with the following profile: 99°C for 1 sec, 59°C for 15 sec, and a final warming to 95°C. Lastly, samples were cooled to 40°C. Samples were loaded in triplicate along with negative controls. Crossing point (CP) values with a standard deviation above 0.5 between replicate samples were dismissed. Negative controls consisted of no template control and were tested on each plate. Primer sequences and annealing temperatures are given in supplementary Table  1. The mRNA expression level for each of the 4 receptors (EGFR and HER2–4) and ligands (amphiregulin HB-EGF and epiregulin) were determined using calibration curves prepared by serial dilution of RNA from cell lines containing the mRNA of interest. The expression levels were normalized to the expression of the reference gene beta-2 microglobulin (B2M).

### 2.5. Western Blotting

Cells were collected in scraping buffer (4 mM iodoacetic acid, 1 mM Na-Orthovanadate, 1 *μ*g/mL of each inhibitor Pepstatin, Chymostatin, Leupeptin, and Aprotinin), centrifuged, resuspended in RIPA buffer (50 nM Tris-HCL pH7.4, 150 nM NaCl, 1% NP-40, 0.25% Na-deoxycholate, 1 mM PMSF, 1 mM Orthovanadate, 1 *μ*g/mL of each inhibitor Aprotinin, Chymostatin, Leupeptin, and Pepstatin), and homogenized with gentle vortexing. Samples were centrifuged at 14,000 g for 15 min at 4°C. The protein concentration was determined using the Nanodrop 2000c (Thermo Scientific). 250 *μ*g proteins were resolved on 4–12% Bis-Tris gels (NuPage, Life Technologies) and transferred to a PVDF membrane (IBlot, Life Technologies). Membranes were blocked using 1X TBST with 5% nonfat dry milk (EGFR, p-EGFR, Akt, Src, p-Scr, MAPK, and p-MAPK) or 5% BSA (p-Akt). For protein detection, the following primary antibodies and dilutions were used: anti-EGFR (Abcam, 1 : 1000), anti-EGFR phospho-Tyr1173 (LSBio, 1 : 500), anti-Akt (Cell Signalling, 1 : 500), anti-Akt phospho-Ser473 (Cell Signalling, 1 : 500), anti-Src (Cell Signalling, 1 : 1000), anti-Src phospho-Tyr416 (Cell Signalling, 1 : 1000), anti-MAPK (Cell Signalling, 1 : 1000), anti-MAPK phospho-Thr202/Tyr204 (Cell Signalling, 1 : 1000), and anti-Histone H3 (Cell Signalling, 1 : 2000). Antibodies were diluted in either 1X TBST with 5% nonfat dry milk (EGFR, p-EGFR, Akt, and H3) or 5% BSA (p-Akt, Src, and p-Src). Goat anti-rabbit secondary antibodies were diluted in 1X TBST with 5% nonfat dry milk (DAKO; EGFR, p-EGFR, Akt, MAPK, and p-MAPK; 1 : 4000) (Cell Signalling; Src, p-Src, and p-Akt; 1 : 5000). SuperSignal West Dura Chemiluminescent Substrate (ECL) was used for detection of protein (Thermo Scientific).

### 2.6. Statistics

The MTS data were analysed by first subtracting the mean background absorbance from each measurement after which the fold changes in cell viability were calculated by the mean absorbance divided by the mean absorbance in the control cell group (not treated) separated by treatment group and cell type. This was done for each experiment separately. Consistency between the experiments was tested by logistic regression analysis and the fold change values were pooled. All cell lines were investigated as triplets or sextuplet. MTS data were reported as mean values with 95% confidence interval for direct comparison and differences in treatment were tested by linear regression.

For each experiment, mean mRNA expression was calculated for the specific receptors and ligands were examined and reported with 95% confidence interval. The mean mRNA expression level was calculated after normalization to a reference gene. Kruskal-Wallis equality-of-populations rank test was used to compare different mRNA expression ratios. Two sided *p* < 0.05 was regarded as significant.

The Western blot and the membrane data were quantified using Image studio Lite v5. The relative density of each band or sport was calculated by using either the control, nontreated group or the control spot as standards. For the statistical analysis STATA (version 13) was used.

## 3. Results

### 3.1. Growth and Sensitivity to Chemotherapy

**Figure 1:**
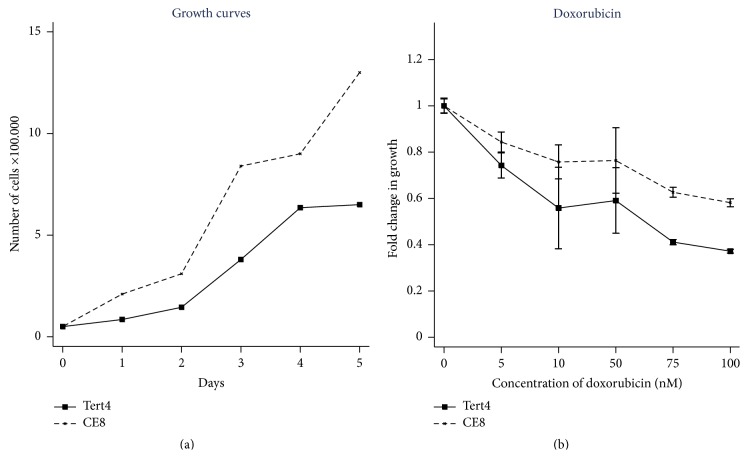
The growth and the sensitivity to doxorubicin of a transformed human mesenchymal (stromal) stem cell line hMSC-TERT4 (solid line) and a derived clonal cell line with the ability to form sarcoma-like tumours in mice hMSC-TERT20-CE8 (CE8, dashed line). The doxorubicin experiments were performed twice with 9 replicates (for the two highest doxorubicin concentrations the experiment was performed once and with 6 replicates). The mean fold change in growth is shown with 95% confidence interval.

The growth of the nontumorigenic hMSC-TERT4 and transformed clonal cell linehMSC-TERT20-CE8 is shown in Figure 1(a). hMSC-TERT20-CE8 had a higher growth rate compared to the nontumorigenic hMSC-TERT4. Cell viability analysis shows that the nontumorigenic hMSC-TERT4 was more sensitive to doxorubicin treatment than hMSC-TERT20-CE8 (Figure 1(b)). Doxorubicin treatment led to decreased cell viability particularly at a dose concentration between 0 and 10 nM.

### 3.2. Pattern of Activated Receptor Tyrosine Kinases

**Figure 2:**
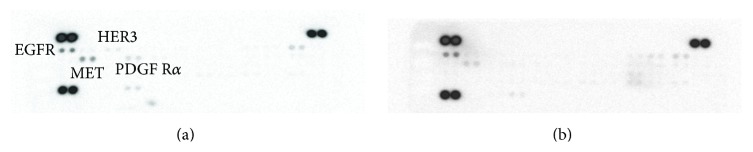
Human Phospho-Receptor Tyrosine Kinase (RTK) Array blots. The activated RTKs are determined in human telomerised stromal stem cell lines (hMSC-TERT). (a) hMSC-TERT4, nontumorigenic. (b) hMSC-TERT20-CE8 a clonal cell line with the ability to form sarcoma-like tumours in mice. The activated tyrosine kinases are represented by black dots on the membranes. For quantification data of the membranes, see supplementary Figure  2 in Supplementary Material available online at http://dx.doi.org/10.1155/2016/9601493.

We employed the RTK array to investigate the RTKs activated in the cell lines. The array detects changes in a panel of 49 RTKs known to be involved in cancer (Figure 2). For both cell lines, EGFR showed a pronounced activation. The MET receptor activity was reduced in hMSC-TERT20-CE8 compared to hMSC-TERT4. Additionally, PDGFR*α* was present in hMSC-TERT4 but with a lower intensity than EGFR. The AXL expression was the same in the two cell lines. For quantification of spots, see supplementary Figure  2.

### 3.3. mRNA Expression of EGF System Receptors and Ligands

To determine the molecular mechanisms of EGFR activation, mRNA expression of the receptors and ligands from the EGF system was determined (Table 1). No difference in expression of EGFR mRNA was found between hMSC-TERT4 and hMSC-TERT20-CE8. The tumorigenic hMSC-TERT20-CE8 showed significantly lower expression of HER2 and HER3 mRNA and a significantly higher expression of the ligands amphiregulin (AR), epiregulin (EPI), and Heparin-binding EGF like growth factor (HB-EGF) compared to the nontumorigenic hMSC-TERT4 (Table 1).

### 3.4. TKI Treatment of the Cells Lines

**Figure 3:**
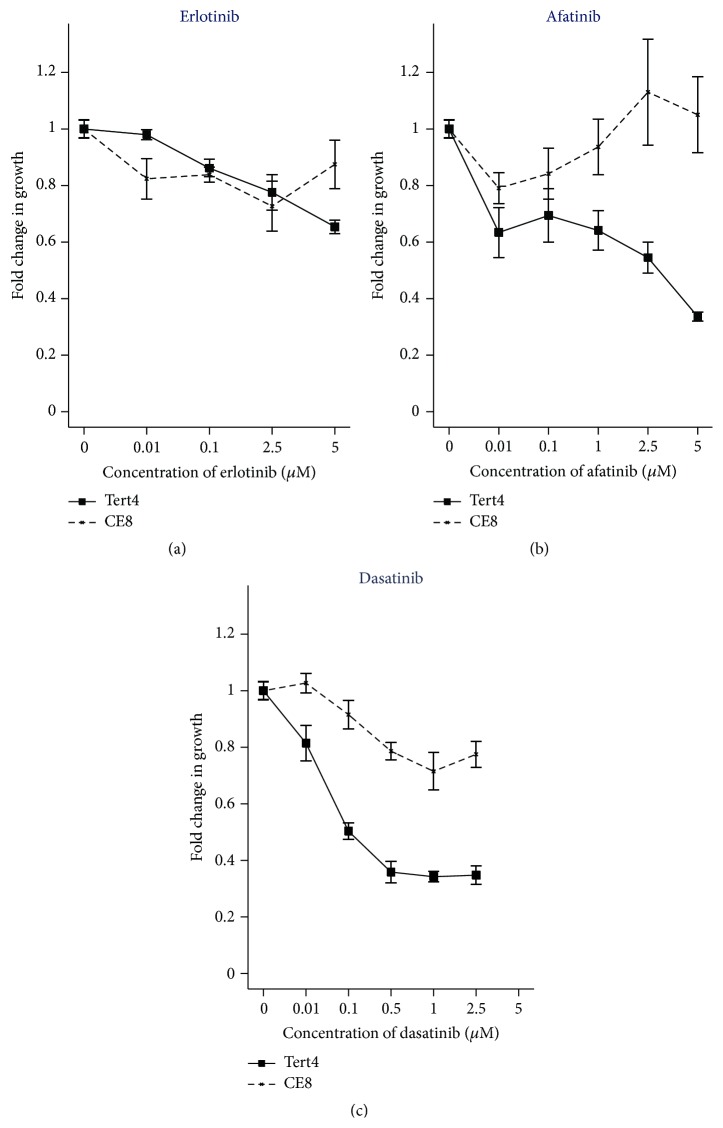
Cell viability was determined by non-Radioactive Cell Proliferation Assay (MTS) for erlotinib (concentrations: 0.01–5 *μ*M), afatinib (concentrations: 0.01–5 *μ*M), and dasatinib (concentrations: 0.01–5 *μ*M) in hMSC-TERT4 (solid line) and hMSC-TERT20-CE8 (dashed lines). The results are presented as fold changes compared to the nontreated cells. The MTS assays were performed twice with total of 9 replicates for the erlotinib treated cells. The MTS assay was performed once with 6 replicates for the afatinib treated cells. The MTS assays were performed two or three times with at least a total of 12 replicates for the dasatinib treated cells. The cell viability is presented as mean value with 95% confidence interval for direct comparison. The cells were treated for 72 hours.

Erlotinib targeting EGFR and afatinib targeting EGFR, HER2, and HER4 were used for testing sensitivity to EGFR system inhibition. The fold change in cell viability, for the hMSC-TERT4 and hMSC-TERT20-CE8 treated with the TKIs, is shown in Figure 3. Erlotinib treatment, at a concentration of 5 *μ*M, decreased cell viability of hMSC-TERT4 to 0.65 (95% CI: 0.63–0.68) and that of hMSC-TERT20-CE8 to 0.88 (95% CI: 0.783–0.96). A significant reduction in cell viability was observed in the hMSC-TERT4 cells treated with erlotinib compared to both the nontreated hMSC-TERT4 (*p* < 0.001) and the erlotinib treated hMSC-TERT20-CE8 cell line (*p* < 0.001).

Afatinib treatment, at a concentration of 5 *μ*M, also showed a change in cell viability of hMSC-TERT4 to 0.34 (95% CI: 0.32; 0.36) and hMSC-TERT20-CE8 to 1.05 (95% CI: 0.87; 1.23). For the hMSC-TERT4 cells, this reduction was significant compared to both nontreated hMSC-TERT4 cells (*p* < 0.001) and afatinib treated hMSC-TERT20-CE8 cells (*p* < 0.001). No significant reduction in cell viability was observed in the afatinib treated hMSC-TERT20-CE8 cells compared to nontreated cells (*p* = 0.28).

**Figure 4:**
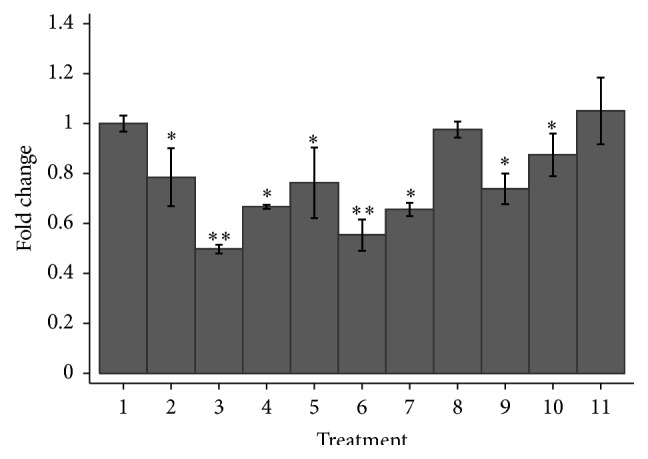
Cell viability expressed as mean fold changes and 95% confidence interval after treatment of hMSC-TERT20-CE8 which are clonal cells lines derived from stromal stem cell line and with the ability to form sarcoma-like tumours in mice. (1) Control, no treatment. (2) Doxorubicin 25 nM. (3) Doxorubicin 25 nM + dasatinib 5 *μ*M. (4) Doxorubicin 25 nM + erlotinib 5 *μ*M. (5) Doxorubicin 50 nM. (6) Doxorubicin 50 nM + dasatinib 5 *μ*M. (7) Doxorubicin 50 nM + erlotinib 5 *μ*M. (8) Doxorubicin 50 nM + afatinib 5 *μ*M. (9) Dasatinib 5 *μ*M. (10) Erlotinib 5 *μ*M. (11) Afatinib 5 *μ*M. The experiments were performed twice with at least a total of 6 replicates of each cell line. Significant results compared to the nontreated cells are marked with *∗* whereas significant results compared to the corresponding doxorubicin treatment are marked with nontreatment *∗∗*.

Combined treatment with the EGFR inhibitors and doxorubicin resulted in no additional effects on hMSC-TERT20-CE8 (Figure 4). These results suggest that direct targeting of EGFR does not reverse the doxorubicin resistance.

**Figure 5:**
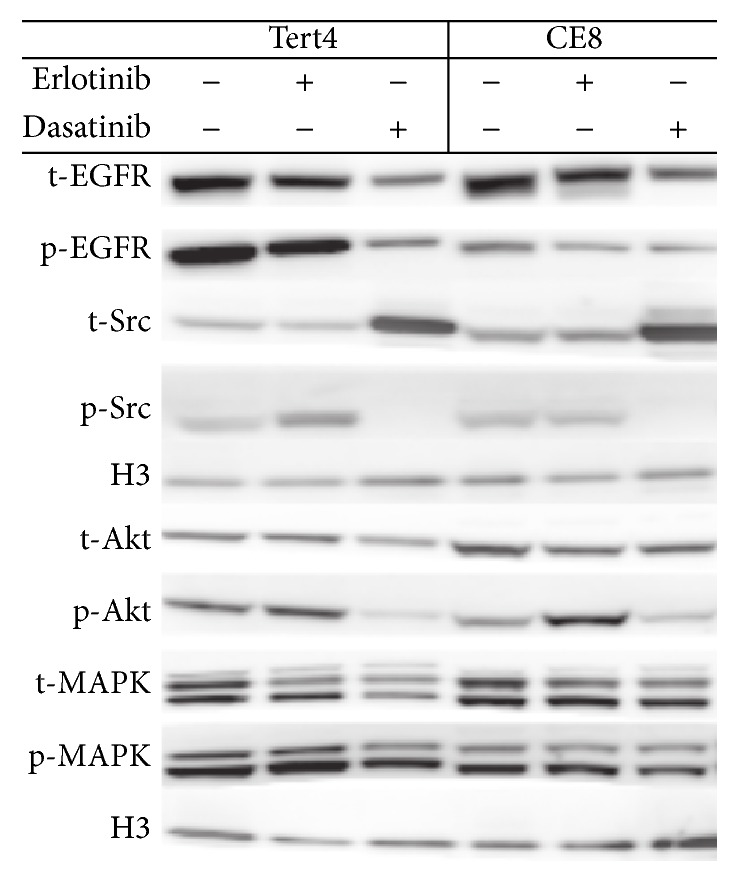
Western blot analysis of total and activation of the EGFR, Src, Akt, and MAPK in hMSC-TERT4 and hMSC-TERT20-CE8 which is clonal cell line derived from mesenchymal (stromal) stem cells and with the ability to form sarcoma-like tumours in mice. The cell lines were treated for 72 h with either vehicle, erlotinib 5 *μ*M, or dasatinib 5 *μ*M. t, total; p, phosphorylated. Histone H3 was used as loading control. For quantification data, see supplementary Figure  3.

We then tested the effect of blocking the downstream tyrosine kinase SRC on the doxorubicin resistance phenotype. Dasatinib is a combined SRC and DDR2 inhibitor. Dasatinib treatment, at a concentration of 5 *μ*M, showed a marked reduction in cell viability of hMSC-TERT4 to 0.28 (95% CI: 0.25; 0.31) and hMSC-TERT20-CE8 to 0.74 (95% CI: 0.77; 0.80). These results were significant when comparing the two treated cells lines (*p* < 0.001) and when compared to the nontreated cells (*p* < 0.001) for each cell line separately. Dasatinib resulted in inhibition of the phosphorylated SRC and AKT pathway, while the MAKP pathway was not affected (Figure 5). For quantitative data on the intensities in the Western blot, see supplementary Figure  3.

The combined treatment with dasatinib (5 *μ*M) and doxorubicin (25 nM) significantly decreased cell viability of hMSC-TERT20-CE8 compared to treatment with doxorubicin alone: 0.50 (95% CI: 0.48; 0.52) versus 0.78 (95% CI: 0.69; 0.90), respectively, *p* = 0.002. The same was true using a doxorubicin concentration of 50 nM (*p* = 0.009).

## 4. Discussion

Our results demonstrated that combining dasatinib and doxorubicin decreases cell viability of a cell line less sensitive to doxorubicin treatment and that targeting EGFR may not be a future treatment strategy for sarcoma patient.

Recent studies have shown that increased expression of EGFR is associated with high-grade sarcoma [11] and poor prognosis [12] and that treatment with EGFR inhibitors can sensitize sarcoma cell lines to chemotherapy in vitro and in vivo [13]. However, a phase II clinical trial treating sarcoma patients resistant to chemotherapy with gefitinib, as single agent therapy, failed in increasing time to tumour progression [14].

The resistance to oncological treatment may reside in a small group of tumour initiating stem cells. Our study used a model of nontransformed and transformed stromal stem cell lines to test the sensitivity of sarcoma tumour initiating stem cells to tyrosine kinase inhibitors. The results of our experiments are in agreement with the clinical results since they showed that the EGFR activated signaling in nontransformed and transformed stromal cell lines did not result in increased sensitivity to treatment with the EGFR inhibitors erlotinib and afatinib. Furthermore, a combined treatment of EGFR inhibitors and doxorubicin did not increase the efficacy of doxorubicin in killing transformed stromal stem cells. The employed erlotinib concentration has previously reduced cell viability in other cancer cell lines [15]. Therefore, even though the reduction in cell viability, comparing nontreated and erlotinib treated hMSC-TERT20-CE8, was significant (*p* = 0.003), the tumorigenic cell line, in this study, is considered relatively resistant to erlotinib.

The erlotinib concentration of 5 *μ*M only reduced activation of EGFR but did not block the activation of EGFR completely. Interestingly, this concentration has been demonstrated to completely inhibit EGFR phosphorylation in other cancer models [16, 17]. This suggests that EGFR in stromal stem cells are cross-activated by other receptors that are refractory to erlotinib inhibition. Our findings correspond to results from a previous study where coactivation of EGFR and an opioid receptor in non-small cell lung cancer has been observed [18]. We have tested higher concentration of erlotinib (supplementary Figure  1). At these higher concentrations, a further reduction in cell viability was observed. Yet, these erlotinib concentrations are not clinically relevant [19].

Gene expression profiling of leiomyosarcomas and undifferentiated pleomorphic sarcomas has suggested that SRC can be employed as a diagnostic marker [20]. SRC is activated through EGFR system or the discoidin domain receptor 2 (DDR2) [21]. DDR2 is a tyrosine kinase (RTK) expressed mainly in stromal or fibroblastic cells. An increased expression and activation of DDR2 in breast cancer cells are associated with a malignant phenotype and the expression of DDR2 is increased as a result of hypoxia [22].

Our study shows that, by inhibiting a downstream RTK of EGFR, using dasatinib, which among other inhibits SRC and DDR2, only reduces cancer cell viability slightly when given alone but significantly decreases cells' viability of the otherwise doxorubicin stem cell line when combined with doxorubicin. Dasatinib inhibits PDGFR, DDR2, and the SRC family. Our results are supported by the work of van Oosterwijk et al. showing that dasatinib acts synergistically with doxorubicin in inhibiting cell viability of chondrosarcoma cell lines [23]. It has previously been shown in breast carcinoma cell lines that inhibition of SRC family kinases prevents the phosphorylation of MAPK induced by stimulation of EGFR [17]. However, our results show that inhibition of SRC did not decrease the phosphorylated state of MAPK, whereas the phosphorylation of AKT was lowered in both the transformed and the nontransformed cell lines.

The transformed cell line was responsive to the combined treatment with doxorubicin and dasatinib. Coactivation of RTKs was tested by using p-AKT protein as a molecular surrogate for downstream RTK signaling. We could confirm that single agent treatment with erlotinib could not effectively block phosphorylation of the AKT protein.

It is believed that most of the tumour volume is composed of descendants from cancer-initiating cells [24]. These heterogeneous clusters of various tumour cells may have different molecular pathways to overcome treatment effects and will therefore have different sensitivities to different treatments. Targeting the downstream pathway of SRC is only one of various possible mechanisms and is not expected therefore to be a universal mechanism to sensitize sarcomas to doxorubicin. Furthermore, dasatinib is not a specific inhibitor of SRC. Indeed another hMSC-TERT derived tumorigenic cell line (BD11) was not responsive to dasatinib (data not shown) and the nontransformed cell line hMSC-TERT4 was even more sensitive to dasatinib than hMSC-TERT-CE8. The activation of critical pathways for survival of mesenchymal cancer cells may be determined by the sum of multiple inputs and multiple RTKs. These RTKs may be active simultaneously or sequentially to maintain cell survival [5]. Therefore, a combination of different TKIs, a combination of TKIs and chemotherapy, or targeting downstream TKIs may be required.

Our study suggest that combining dasatinib and doxorubicin results in lower cell viability than either treatment alone. Therefore, this may be a treatment modality to be considered for metastatic STS patients. We also propose that different resistant cell lines acquire different resistant mechanisms even though they originated from the same parental stem cell.

## Supplementary Material

1. Supplementary Table 1: gene assays.
2. Supplementary Figure 1: Fold change in growth at different doxorubicin and erlotinib concentrations.
3. Supplementary Figure 2: Quantification of activated RTK.
4. Quantification of Western blot.

## Figures and Tables

**Table 1:** The mean mRNA expression ratio of HER1, HER2, HER3, and HER4 receptors for the EGF system and the ligands AR, EPI, and HB for the parental cell line hMSC-TERT4 and the derived clonal cell line hMSC-TERT20-CE8 with the ability to form sarcoma-like tumours in mice. All expressions levels are normalized to reference gene B2M. The numbers in bold represent the gene that exhibits significant changes when comparing hMSC-TERT20-CE8 with the parental cell line hMSC-TERT4.

Gene	Mean expression ratio (95% CI)					
	hMSC-TERT4			hMSC-TERT20-CE8		
	*n*	Mean	95% CI	*n*	Mean	95% CI
HER1	6	3.11	(2.05; 4.17)	9	2.58	(2.12; 3.04)
HER2	6	4.59	(3.65; 5.53)	9	**0.93**	(0.66; 1.19)
HER3	9	0.76	(0.63; 0.88)	12	**0.43**	(0.32; 0.54)
HER4	6	0.10	(0.00; 0.19)	9	**0.03**	(0.01; 0.05)
AR	6	0.65	(0.43; 0.86)	9	**40.09**	(31.09; 49.01)
EPI	6	0.06	(0.00; 0.12)	9	**28.64**	(17.28; 39.99)
HB	6	6.65	(3.76; 9.54)	9	**21.52**	(13.27; 29.77)
