# Supplementary material for: Dasatinib and Doxorubicin Treatment of Sarcoma Initiating Cells: A Possible New Treatment Strategy
Source: Stem Cells Int. 2015 Dec 15;2016:9601493. doi: 10.1155/2016/9601493 (PMC4693025; doi:10.1155/2016/9601493)

Supplementary table I

Assay	Forward primer	Reverse primer	Annealing temp
EGFR	5'-GAG AAC GCC TCC CTC A-3'	5'-GGT ACT CGT CGG CAT C-3'	54
HER2	5'-CCA GGA CCT GCT GAA CTG CT-3'	5'-TGT ACG AGC CGC ACA TCC-3'	59
HER3	5'-AGG GAC CCA GGT CTA CGA TG-3'	5'-CTC ACT ATG TCC CTC CAG TC-3'	60
HER4	5'-ACA GCA GTA CCG AGC CTT TGC G-3'	5'-GCC ACT AAC ACG TAG CCT GTG AC-3'	64
AR	5'-GCC TCA GGC CAT TAT GC-3'	5'- ACC TGT TCA ACT CTG ACT GA-3'	58
HB-EGF	5'-GGT GGT GCT GAA GCT CTT TC-3'	5'-CCC CTT GCC TTT CTT CTT TC-3'	61
EPI	5'-CAA AGT GTA GCT CTG ACA TG-3	5'-CTG TAC CAT CTG CAG AAA TA-3'	60

probe HER2: 5'-CAG ATT GCC AGG GGG ATG AGC TAC CTG-3'

Supplementary figure I The growth and the sensitivity to doxorubicin of a transformed human mesenchymal (stromal) stem cell line hMSC-TERT4 (solid line) and a derived clonal cell line with the ability to form sarcoma-like tumours in mice hMSC-TERT20-CE8 (CE8 – dashed line). The doxorubicin experiments were performed with 6 replicates. The mean fold change in growth is shown with 95% confidence interval.

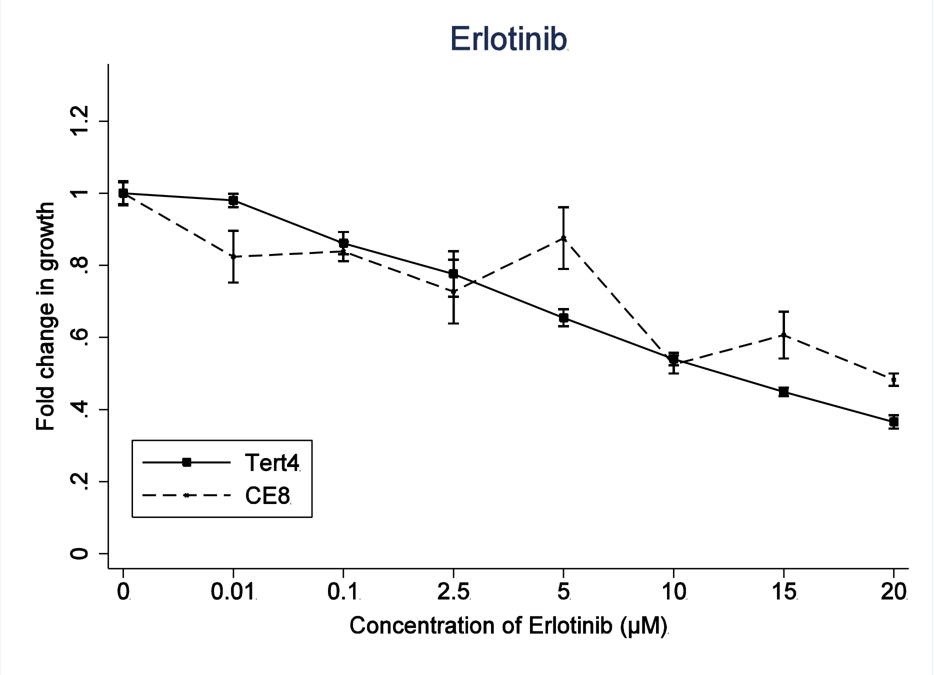

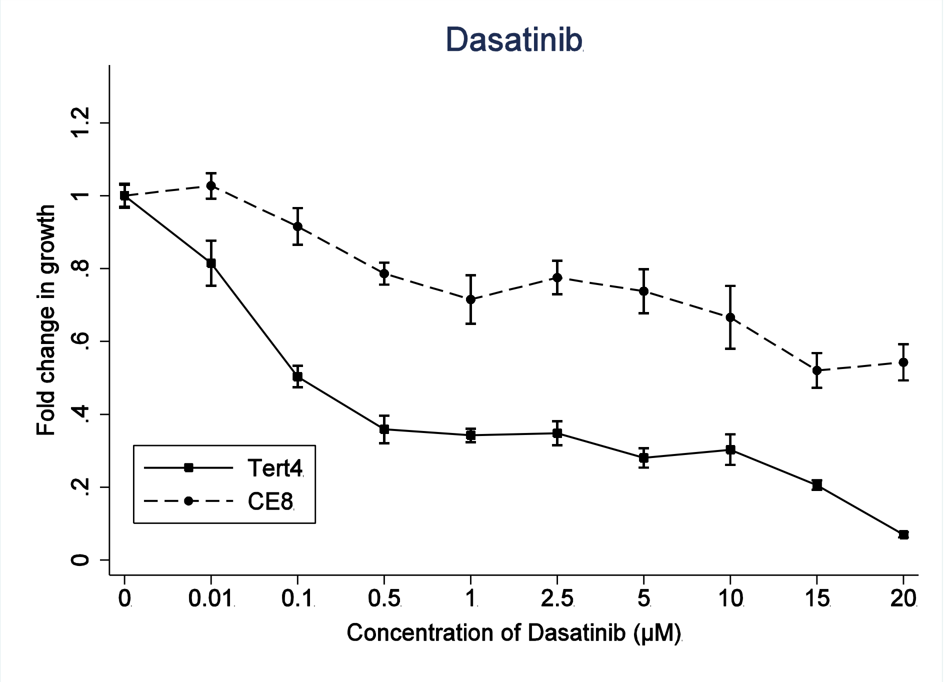

Supplementary figure II Quantification of the activated RTK (figure 2) for hMSC_TERT4 and hMSC-TERT20-CE8. The relative density for each spot was calculated using the control spot as reference.

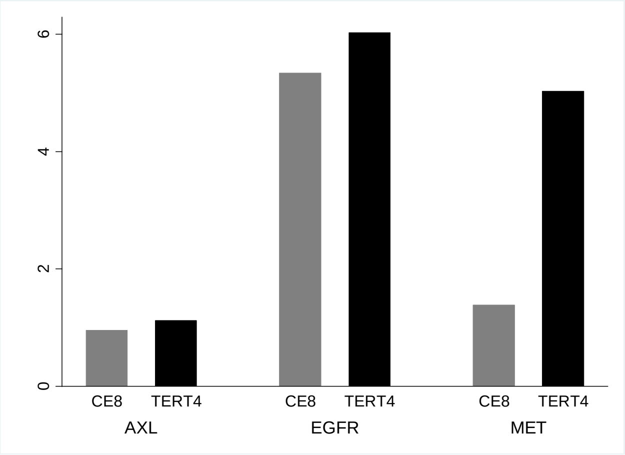

Supplementary figure III Quantification of Western blot figure 5. The total (t) and the activated form (p) of EGFR, Src, Akt and MAPK in hMSC-TERT20- CE8 and hMSC –TERT4 were quantified. The cell lines were treated for 72h with vehicle, erlotinib 5µM or dasatinib 5µM. The relative densities were calculated by using the non treated band on the western blot as reference. Black bars represent hMSC-TERT4 and gray bars represent hMSC-TERT-CE8.

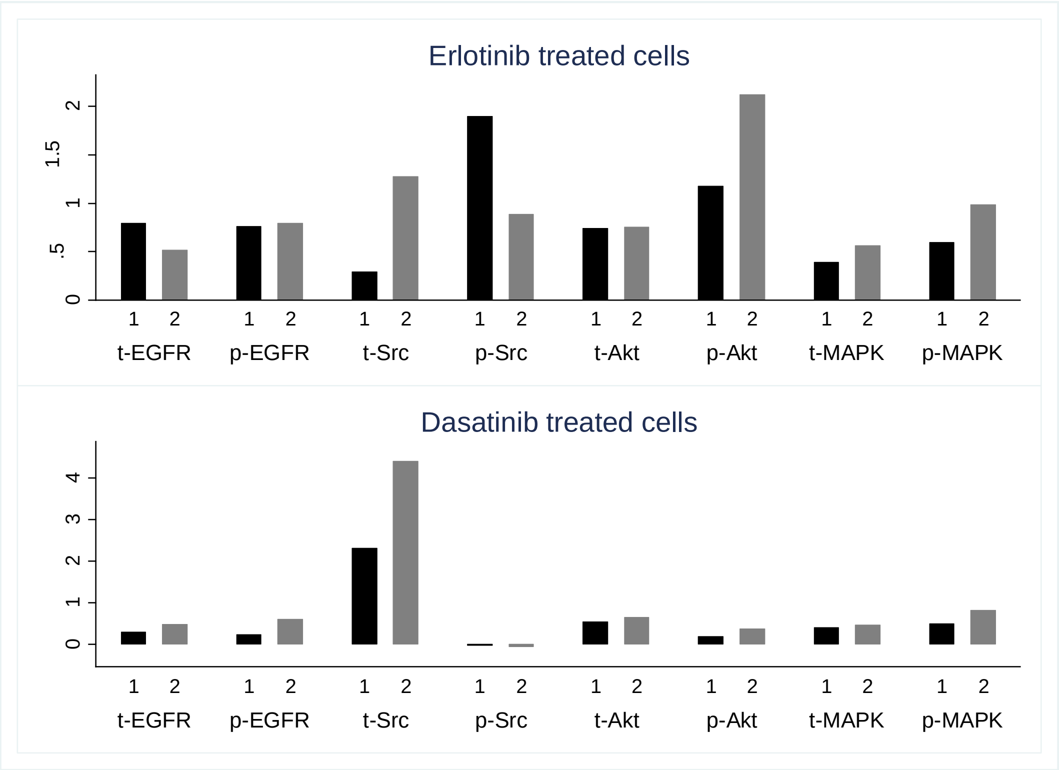

Supplement: Supplementary file 1 — 1. Supplementary Table 1: gene assays. 2. Supplementary Figure 1: Fold change in growth at different doxorubicin and erlotinib concentrations. 3. Supplementary Figure 2: Quantification of activated RTK. 4. Quantification of Western blot. [file 9601493.f1.pdf]
